# Evolution of energy metabolism, stem cells and cancer stem cells: how the Warburg and Barker hypothesis might be linked

**DOI:** 10.1186/1753-6561-7-S2-K8

**Published:** 2013-04-04

**Authors:** James E Trosko

**Affiliations:** 1 Department Pediatrics/Human Development, College of Human Medicine, Michigan State University, East Lansing, Michigan 48824, USA

## 

Two explanations for the origin of cancers exist: the “*Stem Cell Theory*”[1-4] or the “*De-Differentiation*” or “*Reprogramming Theory”*[5]. Concepts related to the genesis of cancers include: (a) The *Multi-Stage*, *Multi-Mechanism* concept of carcinogenesis [6]; (b) the evolution of earth’s physical environment ultimately allowed the appearance of anaerobic microbiological life forms that metabolized via glycolysis [7]; (b) the evolution of photosynthetic algae led to the oxygenation of the environment and to proto-eukaryotes after the symbiotic marriage of bacteria that could produce energy via oxidative phosphorylation; (d) the *Warburg* metabolism of cancers [8]; (e) the concept of “*cancer stem cells*” and “*cancer non-stem cells”*[9]; and (f) the *Barker* hypothesis (diseases later in life might be the result of in utero embryonic/fetal exposures to a variety of factors [10]). To prevent and treat cancer, one must understand the complex mechanism of the multi-stage, multi-mechanism process of human carcinogenesis [11]. Starting with the **initiation** step of transforming a normal cell to one that is unable to terminally differentiate, and the **“promotion”** phase which comes about by the clonal expansion of this single initiated cell. Promotion is brought about the reversible inhibition of **gap junctional intercellular communication**, caused by growth factors, inflammatory stimulation, compensatory hyperplasia, due to chronic irritation or cell death, and by the ability to resist apoptotic death. A single cell can, then, accrue enough genotypic and epigenetic alterations to acquire all the “hallmarks” [12] of an invasive and metastatic cancer cell (the “**progression**” phase). Two important questions arise from this concept of the initiation/promotion/ progression process of carcinogenesis, namely; “*What is the cell that is the target cell for ‘initiation’?*”, and “*What are the underlying molecular mechanisms for each phase of carcinogenesis?*” With the discovery of cancer- initiating cells within a tumor, the concept of **“cancer stem cells”** has been generated. Implicit in this concept is the idea that each tumor is a heterogeneous mixture of **“cancer non-stem cells”** and **“cancer stem cells”**. The current paradigm is based on the assumption that a somatic mortal, differentiated cell can be de-differentiated or re-programmed to become immortal, allowing it to survive long enough to accrue additional mutations and epigenetic changes to become neoplastically-transformed. This paradigm is supported by observations in the stem cell field, where **“induced pluripotent stem cells” (“iPS”)** can be isolated from primary in vitro cultures with various cocktails of embryonic “stemness” genes [13]. However, there is an alterative interpretation of the origin of these “iPS” cells, namely, they were selected adult stem cells in those primary cultures [9]. In addition, the isolation of “MUSE” cells from normal human skin has demonstrated that these rare cells in the skin are the “target cells” for the so-called “iPS” cells [14]. Normal human adult stem cells are naturally “immortal” until they are induced to terminally differentiate. Adult stem cells can be inhibited from “mortalizing” or to remain “immortal” [15]. Dramatic demonstration has shown that only normal human breast stem cells could be efficiently blocked from “mortalization” and then neoplastically-transformed. This observation strongly supports the stem cell hypothesis [16,17]. **Individual genetic, gender, dietary, environmental, life style, medical, lifespan and cultural factors** can affect each of these three phases of carcinogenesis [18-21]. Genetic predispositions, such as xeroderma pigmentosum, leading to UV-induced skin cancer and the experimental carcinogenesis studies and epidemiological findings that clearly show how diets, environmental chemicals (asbestos), drugs (DES; estrogens), life style factors (alcohol; cigarette smoking) can enhance the risk to various cancers. What is sometimes ignored are cultural factors, such as postponement of childbearing (early child bearing is a cancer risk reducer). Other cultural factors, e.g., reduced exercise and dramatically altered diets and nutrition, have been associated with an increased caloric intake and non-nutritional diets of processed foods. A collision of biological evolutionary with cultural evolution is occurring. This allows for increased caloric intake, change in eating habits, types of foods, and even the relationship of the biological evolutional symbiotic role of our gut microbiota to our gut biology [22]. This collision has been more pronounced with caloric over-abundance, dramatically less physical exercise, the eating of processed foods and less of the early human diet-related foods, more grilled red meat, eating at all hours of the day, the changing of our gut microbiota and dieting with supplements. Clearly, biological evolution does not work fast enough to keep up with cultural evolutionary changes that affect our diet and other life style changes (postponing marriage; living longer). While all those factors can influence any of the phases of the carcinogenic process, initiation can **never be reduced to a zero risk**. Every time any cell replicates, there is always a finite chance of a mutation/initiation event to occur. The longer we live, the more initiated cells we accumulate. Except for teratomas, and early childhood cancers, most adult cancers take decades for the promotion process to expand the numbers of initiated cells for more mutational and epigenetic events to occur. Therefore, **the promotion** phase is the **most efficacious period to intervene to prevent** many cancers associated with environmental, dietary, life style, exercise and other cultural factors. One of the newer concepts that might also influence our understanding of risk factors to cancers is the “**Barker hypothesis**”. Indirect experimental studies, as well as epidemiological studies, suggest that *modulation* (*increased or decreased*) *of organ-specific adult stem cells* could increase or decrease the risks to organ-specific cancers later in life ( e.g., DES-exposed female fetuses led to vaginal cancers in young women; soy- and caloric- restricted female fetuses of Japanese women have been associated with low breast cancer frequencies of woman later in life) [22]. Two factors could reduce the risks to various cancers, namely, **modulating stem cell numbers in utero** by careful exposures to environmental/life style and nutritional factors (decrease the target size of ‘initiation’ step) and post-natally, by interfering with the “promotion” of initiated cells by those same factors.

## Competing interests

There are no competing interests in this presentation.
